# 基于新型细胞死亡方式的肺癌预后模型与临床转化前景

**DOI:** 10.3779/j.issn.1009-3419.2026.101.19

**Published:** 2026-08-20

**Authors:** Linjie SUN, Yunkai YOU, Ying ZHANG, Xintong JIANG, Shuyang XIE, Pingyu WANG

**Affiliations:** 264003 烟台，山东医药大学; Shandong Medical and Pharmaceutical University, Yantai 264003, China

**Keywords:** 肺肿瘤, 铁死亡, 铜死亡, 双硫死亡, 钠过载细胞死亡, 预后模型, Lung neoplasms, Ferroptosis, Cuproptosis, Disulfidptosis, Necrosis by sodium overload, Prognostic model

## Abstract

肺癌作为全球发病率和死亡率均居高位的恶性肿瘤之一，其高度异质性和治疗耐药性严重影响了肺癌患者的预后。近年来，铁死亡、铜死亡、双硫死亡、钠过载细胞死亡等新型细胞死亡方式，已成为肿瘤研究热点。本综述围绕上述4类细胞死亡方式，系统梳理其在肺癌发生发展、肿瘤微环境调控和治疗耐药中的作用，归纳相关预后标志物及代表性预后模型，并总结多种死亡方式的交互机制与临床转化进展，以期为完善肺癌预后评估、解析耐药机制及开发新型治疗策略提供参考。

## Abstract (en)

As one of the malignant tumors with high incidence and mortality worldwide, lung cancer features high heterogeneity and therapeutic resistance, which severely impair the prognosis of patients with lung cancer. In recent years, novel cell death modes such as ferroptosis, cuproptosis, disulfidptosis and necrosis by sodium overload have become research hotspots in oncology. Focusing on these four forms of cell death, this review systematically clarifies their roles in lung cancer initiation and progression, regulation of the tumor microenvironment, and therapeutic resistance; summarizes related prognostic biomarkers and representative prognostic models; and discusses the crosstalk among multiple cell death pathways and advances in clinical translation. This review aims to provide a reference for improving prognostic assessment in lung cancer, elucidating mechanisms of therapeutic resistance, and developing novel antitumor strategies.

肺癌是全球新发病例数和死亡例数均居首位的恶性肿瘤^[1]^。肿瘤原发灶-淋巴结-转移（tumor-node-metastasis, TNM）分期、组织学类型及表皮生长因子受体（epidermal growth factor receptor, EGFR）、间变性淋巴瘤激酶（anaplastic lymphoma kinase, ALK）等分子标志物构成当前预后判断和治疗选择的基础依据^[2-4]^。然而，相同分期或驱动基因状态的患者仍可出现不同的疾病进程、耐药演化和生存结局；这一差异不仅取决于肿瘤的遗传背景，还与肿瘤细胞在缺氧、营养限制、氧化应激和药物压力下维持代谢、氧化还原及离子稳态的能力密切相关。上述适应过程共同决定细胞跨越死亡阈值的难易程度，进而影响肿瘤进展、治疗反应和患者预后。因此，围绕细胞死亡调控机制的研究可进一步解析传统分期和驱动基因之外的功能异质性，并为预后标志物和治疗靶点的发现提供新的切入点。

近年来，铁死亡（ferroptosis）、铜死亡（cuproptosis）、双硫死亡（disulfidptosis）和钠过载细胞死亡（necrosis by sodium overload, NECSO）相继被揭示^[5-8]^。这些细胞死亡方式从不同层面刻画了肺癌细胞的死亡脆弱性：铁死亡反映脂质过氧化与抗氧化防御之间的失衡，铜死亡揭示线粒体脂酰化蛋白毒性，双硫死亡体现葡萄糖受限条件下的二硫化物应激，NECSO则将Na^+^过载与线粒体能量崩溃相联系。相关分子特征可用于构建预后模型，解析肺癌细胞在不同微环境和治疗压力下的适应状态，评估患者的代谢应激、免疫微环境及潜在治疗反应。本文围绕4类死亡方式的关键机制、代表性模型、相互作用和临床转化进展展开综述，全文框架见图1。

**图1:**
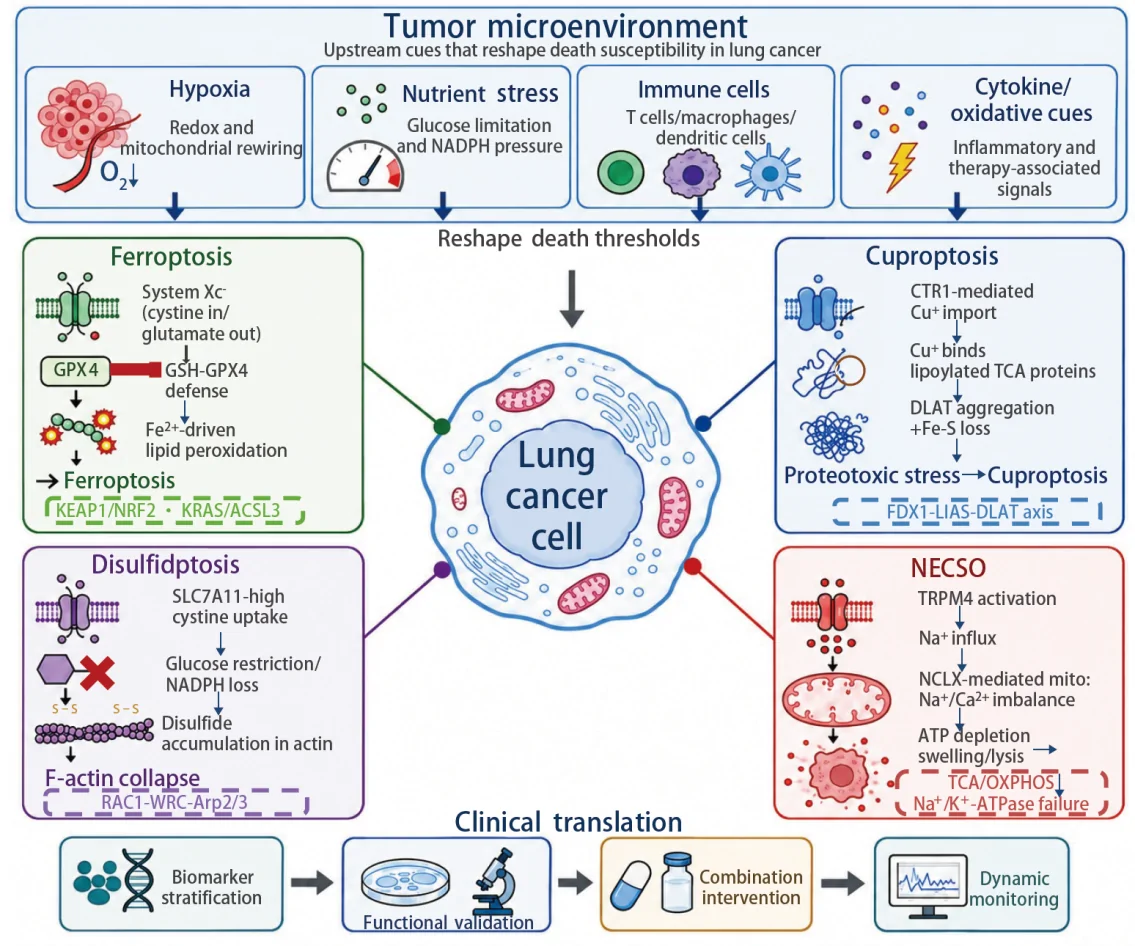
铁死亡、铜死亡、双硫死亡、钠过载细胞死亡在肺癌中的机制与临床转化前景

## 1 铁死亡与肺癌

铁死亡是由铁依赖性膜脂过氧化超过细胞抗氧化清除能力所触发的受调控细胞死亡，其执行与易氧化膜脂、铁代谢、氧化还原防御及膜稳态调节密切相关^[9]^。在肿瘤中，铁死亡阈值又受到驱动基因背景、肿瘤微环境和治疗压力共同塑造，并参与肿瘤抑制、免疫调控和治疗反应^[10]^。

### 1.1 关键机制及肺癌研究进展

肺癌细胞对铁死亡的敏感性首先取决于易过氧化磷脂的供给：Kirsten大鼠肉瘤病毒癌基因同源物（Kirsten rat sarcoma viral oncogene homolog, *KRAS*）突变可通过ACSL3依赖的脂质代谢重塑改变膜磷脂组成，从而影响铁死亡敏感性；相应地，抑制脂质合成或改变磷脂重塑可增强*KRAS*突变肺癌细胞对铁死亡的易感性^[11,12]^。在底物形成后，溶质载体家族7成员11（solute carrier family 7 member 11, SLC7A11）介导胱氨酸摄取并支持谷胱甘肽（glutathione, GSH）合成，谷胱甘肽过氧化物酶4（glutathione peroxidase 4, GPX4）则清除磷脂过氧化物，构成铁死亡防御的重要抗氧化屏障；TRIM3促进SLC7A11蛋白降解，RBMS1调节其翻译^[13,14]^，而*KEAP1*缺失或NRF2异常激活可增强抗铁死亡防线并参与治疗抵抗^[15,16]^。因此，脂质底物端与抗氧化防御端并非彼此独立，两者共同决定肺癌细胞能否跨越脂质过氧化阈值，也解释了铁死亡相关分子为何同时关联肿瘤进展、耐药和患者结局。

### 1.2 预后标志物、模型及转化证据

SLC7A11、GPX4和SDCBP2是较具代表性的铁死亡相关标志物，分别连接胱氨酸利用^[17,18]^、脂质过氧化清除^[19,20]^以及肿瘤增殖和免疫状态^[21]^。早期模型多从预定义基因集中进行生存筛选，再以*LASSO*/*Cox*压缩特征，Li等^[22]^从717个铁死亡相关基因出发，利用WGCNA识别相关模块，经单因素*Cox*筛选候选标志物，再比较10类生存学习方法组成的101种模型，最终获得Step *Cox*联合随机生存森林的23基因特征，并在GSE13213中完成外部验证。建模由单一特征压缩拓展至多队列、网络筛选和算法比较，提高了风险特征的稳定性及其对肺腺癌（lung adenocarcinoma, LUAD）和肺鳞癌（lung squamous cell carcinoma, LUSC）的适用性。转化方面，血清SLC7A11已在晚期非小细胞肺癌（non-small cell lung cancer, NSCLC）前瞻性队列中显示预后价值^[23]^，靶向铁死亡抑制蛋白1（ferroptosis suppressor protein 1, FSP1）则可在多种肺癌模型中诱导铁死亡并抑制肿瘤生长^[24]^。

## 2 铜死亡与肺癌

铜死亡是一种由铜与三羧酸循环脂酰化蛋白直接结合所引发的线粒体蛋白毒性死亡，其独特性在于铜稳态、蛋白脂酰化和线粒体呼吸必须同时处于适宜背景^[25]^。与前述脂质过氧化不同，铜死亡更直接地反映肺癌细胞对线粒体代谢及铜转运调节的依赖。

### 2.1 关键机制及肺癌研究进展

铜死亡的发生从铜负荷形成，到脂酰化蛋白聚集，出现应激适应。铜转运蛋白1（copper transporter 1, CTR1）介导铜摄取，ATP7A和ATP7B负责铜外排，三者首先决定细胞内可利用铜水平^[25]^；进入细胞的铜在铁氧还蛋白1（ferredoxin 1, FDX1）参与下被还原，并作用于由硫辛酸合成酶（lipoic acid synthetase, LIAS）、二氢硫辛酰胺脱氢酶（dihydrolipoamide dehydrogenase, DLD）及丙酮酸脱氢酶E1亚基α/β（PDHA1/PDHB）维持的脂酰化底物，促使二氢硫辛酰胺S-乙酰转移酶（dihydrolipoamide S-acetyltransferase, DLAT）等蛋白聚集，并伴随铁硫簇蛋白丢失。肺癌细胞可进一步通过降低执行敏感性或增强铜外排实现适应：OTUB1/CDYL-SOX18轴抑制FDX1转录，O8G修饰的circKIAA1797限制铜死亡，ATP7B升高则与乏氧诱导因子-1（hypoxia-inducible factor-1, HIF-1）相关免疫治疗抵抗相联系^[26-28]^。从机制链条看，铜死亡从铜代谢异常逐级传递至线粒体蛋白毒性，并通过FDX1抑制和ATP7B外排形成耐药出口，这也为铜离子载体或双硫仑类药物逆转治疗抵抗提供了机制基础。

### 2.2 预后标志物、模型及转化证据

铜死亡相关预后研究已扩展至蛋白编码基因、长链非编码RNA（long non-coding RNA, lncRNA）和跨死亡方式评分^[29-31]^。LUAD研究多基于蛋白编码基因构建预后特征，并进一步关联免疫状态和药物敏感性；LUSC研究则更常采用铜死亡相关lncRNA描述放疗或药物反应，但其外部验证和机制实验相对有限。这些结果表明，不同组织学亚型的铜死亡特征在变量类型和临床解释上并不一致。转化方面，二乙基二硫代氨基甲酸铜[copper(II) bis(diethyldithiocarbamate), CuET]可增强奥希替尼疗效，并已在耐药细胞、*EGFR*突变患者来源类器官和异种移植模型中获得验证^[32]^；双硫仑联合化疗的IIB期研究还提供了早期人体信号^[33]^。

## 3 双硫死亡与肺癌

双硫死亡主要发生于SLC7A11高表达且处于葡萄糖受限状态的细胞中，其核心结果是异常二硫键在肌动蛋白网络中积累并导致细胞骨架塌陷^[34]^。这一机制表明原本用于维持氧化还原稳态的抗氧化适应可在特定营养条件下转化为细胞死亡易感性。

### 3.1 关键机制及肺癌研究进展

肺癌实体瘤中的灌注不足、营养竞争和区域性葡萄糖匮乏为双硫死亡提供了与体外营养充足条件不同的微环境背景。*KEAP1*缺失通过NRF2持续上调SLC7A11，使胱氨酸摄取维持在较高水平；当葡萄糖转运受抑、磷酸戊糖途径难以持续再生还原型烟酰胺腺嘌呤二核苷酸磷酸（nicotinamide adenine dinucleotide phosphate, NADPH）时，摄入的胱氨酸不能被充分还原，二硫化物因而逐渐积累^[35]^。这一代谢压力最终作用于RAC1-WAVE调控复合体（WAVE regulatory complex, WRC）维持的肌动蛋白结构，使蛋白二硫键化、纤维收缩和骨架塌陷依次发生；此外，虽然葡萄糖转运蛋白-1（glucose transporter-1, GLUT1）不是双硫死亡的直接执行者，但其介导的葡萄糖摄取与还原稳态维持密切相关，葡萄糖供应不足时细胞双硫死亡易感性升高^[36]^。

由此，前文所述代谢适应机制呈现出明确的情境依赖性：营养充足时，它通过支持GSH合成抑制铁死亡；葡萄糖受限时，持续胱氨酸输入反而增加NADPH负担并促进双硫死亡，这一情境转换反映了氧化还原适应与营养压力之间的动态平衡。这种作用并不限于肿瘤细胞，信号转导和转录活化因子3（signal transducer and activators of transcription 3, STAT3）-乳酸脱氢酶B（lactate dehydrogenase B, LDHB）轴通过抑制G6PD活性可耗竭肿瘤浸润CD8^+^ T细胞的NADPH，促使其发生双硫死亡并走向耗竭^[37]^；与此同时，LUAD细胞中沉默*ZIC5*可抑制糖酵解并增强双硫死亡^[38]^。因此，糖代谢干预能否获得治疗收益取决于其对肿瘤细胞和效应T细胞的相对影响，患者筛选、肿瘤定向递送及给药时序将共同决定治疗窗口。

### 3.2 预后标志物、模型及转化证据

双硫死亡相关模型在LUAD和LUSC中呈现出不同特征。LUAD的4个蛋白编码基因模型完成了独立基因表达综合（Gene Expression Omnibus, GEO）数据库验证，并围绕PPP1R14B开展细胞功能实验^[39]^；LUSC的lncRNA模型报告了较高的3年和5年曲线下面积（area under the curve, AUC），但验证主要限于TCGA内部队列，实验集中于侵袭和迁移表型^[40]^。前者以蛋白编码基因表征风险并具有外部和功能验证，后者以lncRNA特征为主且证据主要来自内部分析。近期研究进一步结合独立队列、临床样本及细胞功能实验强化模型验证^[41]^。现有模型在变量类型、验证层级和营养背景上仍存在明显差异，LUAD与LUSC对双硫死亡的易感性尚需在统一SLC7A11背景和营养条件下进行比较。

## 4 NECSO与肺癌

NECSO是由瞬时受体电位M亚族成员4（transient receptor potential melastatin 4, TRPM4）持续开放和细胞内Na^+^过载触发的受调控坏死，其执行过程将离子梯度、线粒体能量生成和膜完整性连接成自我放大的失衡环。与前3类死亡方式相比，NECSO的直接机制证据较新，因此肺癌研究需要特别区分离子稳态相关性、TRPM4共表达特征与真正的Na^+^过载执行事件。

### 4.1 关键机制及肺癌研究进展

TRPM4是Ca²^+^激活、ATP抑制的单价阳离子通道。Necrocide-1（NC1）持续激活TRPM4后诱导Na^+^内流，随后通过线粒体Na^+^/Ca²^+^/Li^+^交换体介导的Na^+^/Ca²^+^交换导致线粒体Na^+^积累和Ca²^+^下降，进而抑制氧化磷酸化和三羧酸（tricarboxylic acid, TCA）循环^[42]^。由于Ca^2+^供给不足削弱TCA脱氢酶活性和氧化磷酸化，ATP生成随之下降；能量不足又使Na^+^/K^+^-ATPase难以维持跨膜梯度，从而促进Na^+^继续积聚，最终导致渗透性肿胀和质膜裂解。由通道持续开放到线粒体失能到钠泵失活到Na^+^进一步积聚构成的正反馈，是NECSO区别于脂质过氧化、蛋白毒性和骨架二硫化物应激的主要执行特征。肺癌中Na^+^/K^+^-ATPase α1相关信号参与免疫逃逸和免疫微环境重塑^[43]^。有研究^[44]^进一步定位了NC1在人TRPM4上的结合位点并阐明其物种选择性，使人源肺癌细胞、类器官及表达人TRPM4的模型成为连接通道结构、离子通量和药效安全性的重要验证平台。在肿瘤治疗研究^[45]^中，TRPM4介导的坏死可伴随Na^+^相关细胞肿胀、ATP耗竭、损伤相关分子释放和髓系细胞激活，为理解TRPM4相关坏死的肿瘤学意义提供了证据。

### 4.2 预后标志物、模型及转化证据

现有LUAD研究多以NECSO相关转录特征为起点，但所获得的信息层级不同。Liang等^[46]^基于NECSO相关共表达基因构建15基因风险评分，并完成外部验证；Shi等^[47]^进一步整合单细胞与bulk转录组，在构建8基因特征的同时定位相关细胞群，并分析拟时序和细胞通信。前者主要在整体组织层面刻画风险，后者进一步解析相关细胞状态及其微环境联系。不过，这些模型捕捉的仍是NECSO相关转录状态，而非Na^+^积聚、线粒体Ca^2+^下降、ATP耗竭和膜裂解等执行事件。后续模型的生物学验证可由转录代理转向细胞状态定位和执行标志物验证方向，将单细胞或空间信息与活细胞Na^+^成像、线粒体Ca^2+^/ATP传感和膜完整性检测结合，建立连续证据链。

## 5 细胞死亡的交互调控、预后评估与临床转化

### 5.1 交互调控与脆弱性转移

4类死亡方式可分别从代谢氧化还原、线粒体功能和离子稳态3个层面理解。SLC7A11-GSH-NADPH轴构成铁死亡与双硫死亡之间较明确的转换节点：营养充足时，SLC7A11介导的胱氨酸摄取支持GSH合成和GPX4抗氧化防御；葡萄糖受限时，持续胱氨酸还原则增加NADPH负担和二硫化物应激。CuET可同时诱导铜死亡和铁死亡相关过程，提示不同死亡方式之间可能存在代谢交叉调控^[48]^。GSH稳态、线粒体代谢和Fe-S簇等共享节点进一步连接铁死亡、铜死亡和双硫死亡的代谢脆弱性^[49]^。NECSO则代表由离子稳态失衡和线粒体能量崩溃驱动的另一类潜在死亡脆弱状态。

### 5.2 预后模型比较与定位

现有肺癌预后模型多以*Cox/LASSO*为基础，并进一步引入网络分析、单细胞整合和多算法比较等方法。常规范式通常从候选特征确定、单因素*Cox*筛选、*LASSO*/*Cox*降维及内部/外部验证，Tian等^[50]^可作为其代表；在此基础上，Wei等^[51]^进一步整合13种程序性细胞死亡模式构建程序性细胞死亡综合指数（programmed cell death index, PCDI）。表1^[22,30,31,39,40,46,47,50,51]^主要呈现各模型相对于常规*Cox*/*LASSO*流程的差异化建模特征，并列出训练集3年总生存期（overall survival, OS）的时间依赖受试者工作曲线AUC及主要验证证据。由于各研究的细胞死亡、数据集、人群构成和验证方案不同，不宜直接进行横向比较。

**表1:** 新型细胞死亡相关肺癌预后模型比较

Study	Research content	Modeling method	3-year OS AUC	Key evidence
Li et al. (2025)^[22]^	Ferroptosis	WGCNA+101 algorithms; Stepwise Cox+RSF	LUAD: 0.620; LUSC: 0.630	External+expression validation
Shen et al. (2023)^[30]^	Ferroptosis+cuproptosis	Consensus clustering+PCA; CuFescore	Not reported	Multicohort analysis
Sun et al. (2023)^[31]^	Cuproptosis	Conventional Cox/LASSO	LUAD: 0.740	External validation
Dong et al. (2025)^[39]^	Disulfidptosis	Multivariable Cox refinement	LUAD: 0.759	External+functional validation
Zhu et al. (2024)^[40]^	Disulfidptosis	Multivariable Cox refinement	LUSC: 0.788	Functional validation
Liang et al. (2026)^[46]^	NECSO	NECSO coexpression screening	LUAD: 0.703	External validation
Shi et al. (2025)^[47]^	NECSO	scRNA-seq+bulk integration; WGCNA	LUAD: 0.675	External+single-cell support
Tian et al. (2021)^[50]^	Ferroptosis	Conventional Cox/LASSO	LUAD: 0.705	External+functional validation
Wei et al. (2023)^[51]^	Multiple PCDs	WGCNA; LASSO+random forest intersection	Not reported	HPA protein expression+treatment association

OS: overall survival; AUC: area under the curve; LUAD: lung adenocarcinoma; LUSC: lung squamous cell carcinoma; WGCNA: weighted gene co-expression network analysis; RSF: random survival forest; PCA: principal component analysis; CuFescore: cuproptosis/ferroptosis score; LASSO: least absolute shrinkage and selection operator; scRNA-seq: single-cell RNA sequencing; NECSO: necrosis by sodium overload; PCDs: programmed cell deaths; HPA: Human Protein Atlas. AUC values are from the training cohorts.

### 5.3 转化证据与临床路径

上述4类死亡方式的转化验证层级各不相同：铁死亡已推进至血清学标志物观察及体内靶向药效验证；铜死亡积累了从耐药细胞、类器官到异种移植模型的连续性药效数据，并伴有早期临床试验信号；双硫死亡已在*KEAP1*缺失模型和患者样本中获得相关性支持；NECSO则明确了人TRPM4的结构基础及线粒体能量崩溃的执行环节。这些证据分别对应可检测标志物、可干预靶点、代谢易感人群和人源药物靶点。临床实施应遵循计算特征发现——模型构建——实验验证——临床验证的逐级递进路径，围绕患者富集、药效学监测和动态复评建立标准化指标。患者来源类器官可作为连接模型预测与治疗决策的重要实验平台，用于辅助药敏评价和联合治疗方案筛选^[52]^。

## 6 瓶颈与展望

### 6.1 临床转化瓶颈与对策

当前瓶颈包括：转录特征与死亡执行缺乏因果闭环；SLC7A11、铜转运体和离子泵亦维持正常组织稳态，靶点特异性不足可能压缩治疗窗；二维培养难以重现缺氧、营养竞争、免疫细胞和药物梯度；固定评分难以反映治疗过程中肿瘤细胞死亡易感性的动态变化，且检测平台、外部验证与报告规范尚未统一^[53,54]^。因此，应首先基于分子分型和执行标志物进行患者筛选，再通过肿瘤定向递送或低剂量联合给药降低正常组织暴露风险；探索先削弱代偿机制、再诱导死亡执行的联合治疗时序，并在患者来源类器官、免疫共培养和体内模型中验证疗效与毒性。模型端需整合纵向多组学、药效指标评价和动态复评，沿独立验证、患者来源模型、剂量/时序优化、早期临床试验推进。

### 6.2 未来展望

预后研究应由单一转录组筛选转向跨组学、跨队列和动态建模。多组学整合可用于联合表征不同分子层面的信息^[55]^；单细胞基础模型可通过多批次整合和迁移学习，为跨数据集的细胞状态表征及后续预后建模提供方法支持^[56]^。空间组学与Perturb-seq有助于定位关键基因的来源细胞并验证因果效应^[57,58]^。在此基础上，应将兼具稳定预测价值和机制依据的基因转化为候选靶点，通过可成药性、治疗窗评价和药物重定位筛选化合物^[59]^，并在耐药模型、患者来源类器官及体内模型中验证死亡执行和联合疗效。细胞死亡理论也将随新方式的发现而拓展：2025年 有研究^[60]^提示先天免疫刺激和代谢扰动可通过线粒体氧化应激、线粒体-质膜接触及局部膜损伤共同驱动质膜裂解。未来应结合时序单细胞、空间多组学、代谢通量、活细胞成像和可控微环境模型，解析缺氧、营养匮乏、炎症、离子变化及治疗压力对死亡阈值、执行顺序和通路转换的影响，识别肺癌亚型特异的干预窗口。

## References

[1] Sung H, Filho AM, Laversanne M, et al. Global cancer statistics 2024: GLOBOCAN estimates of incidence and mortality worldwide for 34 cancers in 186 countries. CA Cancer J Clin, 2026, 76(4): e70090. doi: 10.3322/caac.70090 PMC13343830 42417444

[2] Nicholson AG, Tsao MS, Beasley MB, et al. The 2021 WHO classification of lung tumors: Impact of advances since 2015. J Thorac Oncol, 2022, 17(3): 362-387. doi: 10.1016/j.jtho.2021.11.003 34808341

[3] Rami-Porta R, Nishimura KK, Giroux DJ, et al. The international association for the study of lung cancer lung cancer staging project: Proposals for revision of the TNM stage groups in the forthcoming (ninth) edition of the TNM classification for lung cancer. J Thorac Oncol, 2024, 19(7): 1007-1027. doi: 10.1016/j.jtho.2024.02.011 38447919

[4] Hendriks LE, Kerr KM, Menis J, et al. Oncogene-addicted metastatic non-small-cell lung cancer: ESMO Clinical Practice Guideline for diagnosis, treatment and follow-up. Ann Oncol, 2023, 34(4): 339-357. doi: 10.1016/j.annonc.2022.12.009 36872130

[5] Dixon SJ, Lemberg KM, Lamprecht MR, et al. Ferroptosis: An iron-dependent form of nonapoptotic cell death. Cell, 2012, 149(5): 1060-1072. doi: 10.1016/j.cell.2012.03.042 PMC3367386 22632970

[6] Tsvetkov P, Coy S, Petrova B, et al. Copper induces cell death by targeting lipoylated TCA cycle proteins. Science, 2022, 375(6586): 1254-1261. doi: 10.1126/science.abf0529 PMC9273333 35298263

[7] Liu X, Nie L, Zhang Y, et al. Actin cytoskeleton vulnerability to disulfide stress mediates disulfidptosis. Nat Cell Biol, 2023, 25(3): 404-414. doi: 10.1038/s41556-023-01091-2 PMC10027392 36747082

[8] Fu W, Wang J, Li T, et al. Persistent activation of TRPM4 triggers necrotic cell death characterized by sodium overload. Nat Chem Biol, 2025, 21(8): 1238-1249. doi: 10.1038/s41589-025-01841-3 39915626

[9] Dixon SJ, Olzmann JA. The cell biology of ferroptosis. Nat Rev Mol Cell Biol, 2024, 25(6): 424-442. doi: 10.1038/s41580-024-00703-5 PMC12187608 38366038

[10] Lei G, Zhuang L, Gan B. The roles of ferroptosis in cancer: Tumor suppression, tumor microenvironment, and therapeutic interventions. Cancer Cell, 2024, 42(4): 513-534. doi: 10.1016/j.ccell.2024.03.011 38593779

[11] Padanad MS, Konstantinidou G, Venkateswaran N, et al. Fatty acid oxidation mediated by acyl-CoA synthetase long chain 3 is required for mutant *KRAS* lung tumorigenesis. Cell Rep, 2016, 16(6): 1614-1628. doi: 10.1016/j.celrep.2016.07.009 PMC4981512 27477280

[12] Bartolacci C, Andreani C, Vale G, et al. Targeting *de novo* lipogenesis and the lands cycle induces ferroptosis in *KRAS*-mutant lung cancer. Nat Commun, 2022, 13(1): 4327. doi: 10.1038/s41467-022-31963-4 PMC9325712 35882862

[13] Wang Z, Shen N, Wang Z, et al. TRIM3 facilitates ferroptosis in non-small cell lung cancer through promoting SLC7A11/xCT K11-linked ubiquitination and degradation. Cell Death Differ, 2024, 31(1): 53-64. doi: 10.1038/s41418-023-01239-5 PMC10781973 37978273

[14] Zhang W, Sun Y, Bai L, et al. RBMS1 regulates lung cancer ferroptosis through translational control of SLC7A11. J Clin Invest, 2021, 131(22): e152067. doi: 10.1172/JCI152067 PMC8592553 34609966

[15] Bi G, Liang J, Zhao M, et al. MiR-6077 promotes Cisplatin/Pemetrexed resistance in lung adenocarcinoma via CDKN1A/cell cycle arrest and KEAP1/ferroptosis pathways. Mol Ther Nucleic Acids, 2022, 28: 366-386. doi: 10.1016/j.omtn.2022.03.020 PMC9035384 35505963

[16] Koppula P, Lei G, Zhang Y, et al. A targetable CoQ-FSP1 axis drives ferroptosis- and radiation-resistance in *KEAP1* inactive lung cancers. Nat Commun, 2022, 13(1): 2206. doi: 10.1038/s41467-022-29905-1 PMC9033817 35459868

[17] Zhai H, Xiang X, Pu J, et al. LncRNA SLC7A11AR promotes lung adenocarcinoma progression by inhibiting ferroptosis via promoting SLC7A11 expression. Int J Biol Sci, 2025, 21(10): 4549-4566. doi: 10.7150/ijbs.112233 PMC12320243 40765833

[18] Huang Z, Chen X, Wang Y, et al. SLC7A11 inhibits ferroptosis and downregulates PD-L1 levels in lung adenocarcinoma. Front Immunol, 2024, 15: 1372215. doi: 10.3389/fimmu.2024.1372215 PMC11035808 38655266

[19] Asakawa A, Kawade G, Kurata M, et al. Stratification of lung squamous cell carcinoma based on ferroptosis regulators: Potential for new therapeutic strategies involving ferroptosis induction. Lung Cancer, 2022, 165: 82-90. doi: 10.1016/j.lungcan.2022.01.012 35101731

[20] Liu W, Zhou Y, Duan W, et al. Glutathione peroxidase 4-dependent glutathione high-consumption drives acquired Platinum chemoresistance in lung cancer-derived brain metastasis. Clin Transl Med, 2021, 11(9): e517. doi: 10.1002/ctm2.517 PMC8473645 34586745

[21] Sun S, Gu Z, Yuan S, et al. SDCBP2 promotes tumor progression and is a novel ferroptosis-related prognostic biomarker in lung adenocarcinoma. Front Immunol, 2025, 16: 1692308. doi: 10.3389/fimmu.2025.1692308 PMC12705533 41409290

[22] Li Z, Chen Y, Hou B, et al. Machine learning constructs a ferroptosis related signature for predicting prognosis and drug sensitivity in lung cancer. Cell Oncol (Dordr), 2025, 48(6): 1971-1986. doi: 10.1007/s13402-025-01121-1 PMC12698758 41123817

[23] Wang S, Jin X, Yang X, et al. The predictive value of ferroptosis marker SLC7A11 in the prognosis of advanced non-small cell lung cancer patients. Clin Biochem, 2025, 138: 110961. doi: 10.1016/j.clinbiochem.2025.110961 40562295

[24] Wu K, Vaughan AJ, Bossowski JP, et al. Targeting FSP1 triggers ferroptosis in lung cancer. Nature, 2026, 649(8096): 487-495. doi: 10.1038/s41586-025-09710-8 PMC12779550 41193800

[25] Xue Q, Kang R, Klionsky DJ, et al. Copper metabolism in cell death and autophagy. Autophagy, 2023, 19(8): 2175-2195. doi: 10.1080/15548627.2023.2200554 PMC10351475 37055935

[26] Zhang R, Li Y, Lu H, et al. OTUB1/CDYL axis-mediated epigenetic repression of SOX18 facilitates lung cancer progression by inhibiting FDX1-dependent cuproptosis. Oncogene, 2026, 45(16): 1453-1470. doi: 10.1038/s41388-026-03748-2 41912773

[27] Xu H, Zhao Q, Cai D, et al. O8G-modified circKIAA1797 promotes lung cancer development by inhibiting cuproptosis. J Exp Clin Cancer Res, 2025, 44(1): 110. doi: 10.1186/s13046-025-03365-z PMC11963662 40176113

[28] Li P, Sun Q, Bai S, et al. Combination of the cuproptosis inducer disulfiram and anti-PD-L1 abolishes NSCLC resistance by ATP7B to regulate the HIF-1 signaling pathway. Int J Mol Med, 2024, 53(2): 19. doi: 10.3892/ijmm.2023.5343 PMC10781418 38186308

[29] Tian Z, Cen L, Hua H, et al. Prognostic and therapeutic potential of copper-induced cell death-related lncRNAs in lung squamous cell carcinoma. Clin Exp Med, 2025, 25(1): 135. doi: 10.1007/s10238-025-01663-6 PMC12048434 40316808

[30] Shen Y, Li D, Liang Q, et al. Cross-talk between cuproptosis and ferroptosis regulators defines the tumor microenvironment for the prediction of prognosis and therapies in lung adenocarcinoma. Front Immunol, 2023, 13: 1029092. doi: 10.3389/fimmu.2022.1029092 PMC9887127 36733399

[31] Sun JH, Zhang HW, Liu HR, et al. Construction of lung adenocarcinoma prognosis model and drug sensitivity analysis based on cuproptosis related genes. Zhongguo Feiai Zazhi, 2023, 26(8): 591-604. 10.3779/j.issn.1009-3419.2023.102.31 PMC10558763 37752539

[32] Gao YB, Xie JM, Yang YN, et al. Induction of cuproptosis enhances sensitivity and overcomes resistance to Osimertinib in lung cancer. Signal Transduct Target Ther, 2025, 10(1): 390. doi: 10.1038/s41392-025-02480-9 PMC12658086 41298384

[33] Nechushtan H, Hamamreh Y, Nidal S, et al. A phase IIb trial assessing the addition of disulfiram to chemotherapy for the treatment of metastatic non-small cell lung cancer. Oncologist, 2015, 20(4): 366-367. doi: 10.1634/theoncologist.2014-0424 PMC4391770 25777347

[34] Liu X, Zhuang L, Gan B. Disulfidptosis: Disulfide stress-induced cell death. Trends Cell Biol, 2024, 34(4): 327-337. doi: 10.1016/j.tcb.2023.07.009 37574347

[35] Koppula P, Olszewski K, Zhang Y, et al. KEAP1 deficiency drives glucose dependency and sensitizes lung cancer cells and tumors to GLUT inhibition. iScience, 2021, 24(6): 102649. doi: 10.1016/j.isci.2021.102649 PMC8193145 34151236

[36] Goodwin J, Neugent ML, Lee SY, et al. The distinct metabolic phenotype of lung squamous cell carcinoma defines selective vulnerability to glycolytic inhibition. Nat Commun, 2017, 8: 15503. doi: 10.1038/ncomms15503 PMC5458561 28548087

[37] Wan J, Shi JH, Shi M, et al. Lactate dehydrogenase B facilitates disulfidptosis and exhaustion of tumour-infiltrating CD8^+^ T cells. Nat Cell Biol, 2025, 27(6): 972-982. doi: 10.1038/s41556-025-01673-2 40461882

[38] Zeng C, Huang D, Wang L, et al. Silencing ZIC5 suppresses glycolysis and promotes disulfidptosis in lung adenocarcinoma cells. Cancer Biol Ther, 2025, 26(1): 2501780. doi: 10.1080/15384047.2025.2501780 PMC12080275 40366858

[39] Dong Y, Zhang Y, Liu H, et al. Prognostic model of lung adenocarcinoma based on disulfidptosis-related genes and analysis of *in vitro* cell experiments for PPP1R14B in the model. Biol Direct, 2025, 20(1): 74. doi: 10.1186/s13062-025-00662-7 PMC12210501 40598552

[40] Zhu A, Zong Y, Gao X. Development of a disulfidptosis-related lncRNA prognostic signature for enhanced prognostic assessment and therapeutic strategies in lung squamous cell carcinoma. Sci Rep, 2024, 14(1): 17804. doi: 10.1038/s41598-024-68423-6 PMC11294474 39090162

[41] Wang Y, Xu Y, Liu C, et al. Identification of disulfidptosis-related subgroups and prognostic signatures in lung adenocarcinoma using machine learning and experimental validation. Front Immunol, 2023, 14: 1233260. doi: 10.3389/fimmu.2023.1233260 PMC10548142 37799714

[42] Qiao Y, Wang J, Wang B, et al. Sodium disrupts mitochondrial energy metabolism to execute NECSO. Nat Commun, 2026, 17(1): 493. doi: 10.1038/s41467-025-67181-x PMC12804697 41390760

[43] Yang K, Li Z, Chen Y, et al. Na, K-ATPase α1 cooperates with its endogenous ligand to reprogram immune microenvironment of lung carcinoma and promotes immune escape. Sci Adv, 2023, 9(6): eade5393. doi: 10.1126/sciadv.ade5393 PMC9916986 36763655

[44] Teixeira-Duarte CM, Fu W, Zeng W, et al. Structural mechanism of Necrocide 1 activation of human TRPM4 that triggers necrosis by sodium overload. Nat Commun, 2026, 17(1): 7939. doi: 10.1038/s41467-026-74814-2 PMC13448252 42342693

[45] Ghosh S, Yang R, Duraki D, et al. Plasma membrane channel TRPM4 mediates immunogenic therapy-induced necrosis. Cancer Res, 2023, 83(18): 3115-3130. doi: 10.1158/0008-5472.CAN-23-0157 PMC10635591 37522838

[46] Liang L, Wang X, Liu H, et al. Construction and validation of a prognostic model for lung adenocarcinoma based on necrotic cell death triggered by sodium overload-related coexpressed genes. Front Genet, 2026, 17: 1763705. doi: 10.3389/fgene.2026.1763705 PMC12989553 41847125

[47] Shi Y, Qu X, Liu D, et al. Integration of single-cell and bulk transcriptomics to explore prognostic and immunotherapeutic characteristics of sodium overload-induced cell death in lung adenocarcinoma. Transl Cancer Res, 2025, 14(10): 6330-6347. doi: 10.21037/tcr-2025-919 PMC12605749 41234862

[48] Zhang J, Lei C, Zheng G, et al. CuET promotes cuproptosis and ferroptosis of lung cancer cells by interacting directly with polyunsaturated phospholipids. iScience, 2026, 29(4): 115438. doi: 10.1016/j.isci.2026.115438 PMC13089065 42006359

[49] Mao C, Jiang D, Koong AC, et al. Exploiting metabolic cell death for cancer therapy. Nat Rev Cancer, 2026, 26(1): 27-45. doi: 10.1038/s41568-025-00879-8 41094043

[50] Tian Q, Zhou Y, Zhu L, et al. Development and validation of a ferroptosis-related gene signature for overall survival prediction in lung adenocarcinoma. Front Cell Dev Biol, 2021, 9: 684259. doi: 10.3389/fcell.2021.684259 PMC8294813 34307361

[51] Wei Q, Jiang X, Miao X, et al. Molecular subtypes of lung adenocarcinoma patients for prognosis and therapeutic response prediction with machine learning on 13 programmed cell death patterns. J Cancer Res Clin Oncol, 2023, 149(13): 11351-11368. doi: 10.1007/s00432-023-05000-w PMC11797341 37378675

[52] Chen Y, Xing H, Yang X, et al. Patient-derived malignant effusion organoids guide tailored therapy and dissect third-generation EGFR-tyrosine kinase inhibitor resistance mechanisms in lung cancer. Cancer Lett, 2026, 645: 218356. doi: 10.1016/j.canlet.2026.218356 41740831

[53] Collins GS, Moons KGM, Dhiman P, et al. TRIPOD+AI statement: Updated guidance for reporting clinical prediction models that use regression or machine learning methods. BMJ, 2024, 385: e078378. doi: 10.1136/bmj-2023-078378 PMC11019967 38626948

[54] Riley RD, Collins GS, Ensor J, et al. Minimum sample size calculations for external validation of a clinical prediction model with a time-to-event outcome. Stat Med, 2022, 41(7): 1280-1295. doi: 10.1002/sim.9275 34915593

[55] Argelaguet R, Arnol D, Bredikhin D, et al. MOFA+: A statistical framework for comprehensive integration of multi-modal single-cell data. Genome Biol, 2020, 21(1): 111. doi: 10.1186/s13059-020-02015-1 PMC7212577 32393329

[56] Cui H, Wang C, Maan H, et al. scGPT: Toward building a foundation model for single-cell multi-omics using generative AI. Nat Methods, 2024, 21(8): 1470-1480. doi: 10.1038/s41592-024-02201-0 38409223

[57] Kleshchevnikov V, Shmatko A, Dann E, et al. Cell location maps fine-grained cell types in spatial transcriptomics. Nat Biotechnol, 2022, 40(5): 661-671. doi: 10.1038/s41587-021-01139-4 35027729

[58] Dixit A, Parnas O, Li B, et al. Perturb-Seq: Dissecting molecular circuits with scalable single-cell RNA profiling of pooled genetic screens. Cell, 2016, 167(7): 1853-1866.e17. doi: 10.1016/j.cell.2016.11.038 PMC5181115 27984732

[59] Subramanian A, Narayan R, Corsello SM, et al. A next generation connectivity map: L 1000 platform and the first 1,000,000 profiles. Cell, 2017, 171(6): 1437- 1452.e17. doi: 10.1016/j.cell.2017.10.049 PMC5990023 29195078

[60] Wang Y, Lu J, Carisey AF, et al. Innate immune and metabolic signals induce mitochondria-dependent membrane lysis via mitoxyperiosis. Cell, 2025, 188(25): 7155-7174.e25. doi: 10.1016/j.cell.2025.11.002 PMC12875127 41317732

